# Supplementary material for: Are carbon emissions trading and green financial instruments synergistic? -Comprehensive quantitative research based on content analysis
Source: PLoS One. 2024 Mar 7;19(3):e0298601. doi: 10.1371/journal.pone.0298601 (PMC10919601; doi:10.1371/journal.pone.0298601)
Supplement: S1 Data — (ZIP) [file pone.0298601.s001.zip › Data/地级市Z_F工作报告.zip!/╦─┤¿/├╝╔╜╩╨/└┤╫╘╣1⁄2╓┌║┼í╛┬φ┐╦╩2╛▌═°í┐_2016.docx]

各位代表：

我代表市人民政府，向大会报告工作，请予审查，并请政协委员提出意见。

“十二五”及2015年工作回顾

“十二五”时期，是眉山发展历程中极不平凡的五年，也是经济社会发展取得新的重大成就的五年。五年来，在省委、省政府和市委的坚强领导下，在市人大、市政协的监督支持下，我们紧紧围绕“工业强市、文化立市、环境兴市、依法治市”的发展战略，全面贯彻中央、省委省政府和市委的决策部署，积极应对各种压力挑战，主动适应经济发展新常态，在爬坡上坎中转型升级，在改革创新中攻坚拔寨，圆满完成了“十二五”规划确定的主要目标任务。

这五年，值得振奋的是，眉山发展动能得到大释放。全市地区生产总值（GDP）五年基本翻番，成功站上千亿级台阶，实现历史性突破；县域经济全部晋位升级；全社会固定资产投资累计完成3750亿元，年均增长19.4 % ；规上工业增加值年均增长14.9 % ；社会消费品零售总额年均增长14.8 % ；民营经济增加值年均增长14 % ；民间投资占全社会投资比重达74 % 。

这五年，值得喜悦的是，眉山经济质效得到大提升。三产增加值年均增长11.7 % ，增速保持全省前列；地方一般公共预算收入83.1亿元，是2010年的3.37倍，在全省排名升2位；金融机构存款余额1443亿元、贷款余额691亿元，分别是2010年的2.35倍、2.59倍，存贷比47.9 % ，提高2.15个百分点；城镇化率42 % ，提高7.9个百分点；省上下达的节能减排任务全面完成。

这五年，值得欣慰的是，眉山民生福祉得到大改善。全市全部财政支出中80 % 以上用于民生，实施民生工程703项，累计投入194亿元；城乡居民人均可支配收入2.65万元、1.28万元，分别是2010年的1.8倍、1.93倍；城乡居民收入差距从2010年的2.23缩小到2.08，农村居民人均可支配收入总量连续七年全省第4位；新增城镇就业15.9万人，累计脱贫22.4万人。

五年来，我们主要做了以下工作：

坚持追赶发展，从速度规模型走向质量效益型，产业体系更加优化。五年来，我们牢固树立加快发展的核心理念，坚持在追赶跨越中缩小差距，在转型升级中提质增效，产业发展逐步迈向中高端。产业结构不断优化。三次产业结构从18.8：54.9：26.3调整为15.4：57.7：26.9。“4 + 4”的现代工业体系初步构建，机械装备、新型建材等四大传统优势产业产值均突破百亿元；高端装备制造、新一代电子信息技术等四大新兴产业产值235亿元，战略性新兴产业占工业经济比重的21 % 。现代服务业提速发展，旅游总收入实现230亿元，服务业增加值在国民经济中的比重提高0.6个百分点，消费对经济增长的贡献率达到27 % ，新增国家4A级旅游景区3个、省级度假区1个。“东坡味道”千亿产业加快发展，总产值实现583亿元。产业质态不断优化。工业经济量质齐升，净增规上企业23户达622户，新增50亿元企业集团1户；工业利润、入库税金分别比2010年增长1.3倍、1.7倍。单位工业增加值综合能耗下降40 % ，淘汰和关闭落后产能企业93户。质量强市成效明显，新增国家地理标志证明商标6件、国家地理标志保护产品7个，建成省知名品牌创建示范区2个。“东坡泡菜”品牌价值突破百亿元。产业布局不断优化。园区成为经济发展的主载体，13个市级重点工业园区全面整合，工业集中度达到64.3 % 。建成3个百亿园区、4个50亿园区，彭山经开区、甘眉园区、视高开发区成功纳入省“51025”重点园区培育范围。彭山青龙物流园区成功创建为省级物流聚集区。都市近郊型现代农业“两城三园七片”强力推进，建成国家级畜禽标准化示范场13个、国家级种业基地2个、国家级现代农业示范区1个。岷江现代农业示范园区成功创建为国家级农业产业化示范基地，眉山成为全省整体推进现代农业的3个示范市之一。

坚持创新发展，从要素驱动型走向创新驱动型，发展活力更加强劲。五年来，我们始终把创新摆在经济社会发展的突出位置，充分发挥创新激励经济增长的乘数效应，经济活力进一步增强。综合改革成效明显。农村改革亮点纷呈，彭山区成功列入全国农村改革试验区，新型经营主体培育深入推进，发展“521”体系经验全省推广，“银会对接”模式全国推广。行政体制改革推进有力，率先在全省建立“三张清单”，清理优化权力事项5158项、责任事项3673项；政府机构改革顺利实施，事业单位分类改革稳步推进。经济体制改革纵深拓展，眉山农商行筹建进展顺利，政府平台融资36.9亿元。商事登记制度改革加快推进，大众创业、万众创新如火如荼，新增各类市场主体81549个。创新驱动成绩斐然。科技创新深入实施，成功创建国家技术创新工程四川省试点示范市，新创建国家高新技术企业56家，建成国家和省重点实验室2个。协同创新推进有力，产学研创新联盟加快建设，与13个高校和科研院所签订合作协议，申请发明专利980项，实施科技成果转化398项、产值400亿元。产业创新成效明显，新建创业创新孵化中心（基地）和聚集区5个，创客空间、创意坊6个，电子商务交易额和网络零售额年均增长超过30 % ，快递业务量年均增长超过60 % 。仁寿县、青神县被评为国家级电子商务进农村综合示范县。开放合作成果丰硕。成眉合作、甘眉合作、雅眉合作全面推进。“中法农业科技园”落户眉山。天府新区上升为国家级新区，眉山片区建成区面积突破15平方公里，入驻企业274户。现代工业新城加快建设，总部经济区顺利推进，累计完成投入760亿元。招商引资“376”战略深入实施，中外系列投资促进活动接连开展，成功引进世界500强、中国500强或投资10亿元以上企业132家，地方股份制银行3家，招商引资省外到位资金连续八年全省第2位，实际利用外资连续五年全省前列，眉山成为全省除成都以外落户世界500强企业最多的市州。

坚持跨越发展，从注重物的城镇化走向注重人的城镇化，发展基础更加牢固。五年来，我们坚持以人的城镇化为核心，强力推进城乡要素自由流动、公共资源均衡配置，跨越发展基础更加坚实。城乡面貌明显变化。创新“四种模式”，加快“四个转变”，实现“四有目标”，五年转变农村居民85.3万人。城镇综合承载能力显著增强，城镇建设累计投入867亿元，新增建成区面积51平方公里，国家新型城镇化综合试点城市申报成功。彭山撤县建区，岷东新区初具规模，眉山大城市格局基本奠定。乡镇建设品质提升，率先在全省实现乡镇总规、控规和城镇形象设计全覆盖，布局合理、梯次分明的新型城镇发展格局逐步形成。新村建设成效明显，建成幸福美丽新村355个。城乡基础明显夯实。综合交通颠覆区位，五年完成投入456亿元，新增公路里程560公里，“两横三纵”高速路网全面建成，“三横九纵”铁路网日益完善，“四箭齐发”加快推进，眉山到成都、到区县“半小时经济圈”全面形成，成功迈入“双高”“双港”时代。乡村道路四通八达，新改建农村公路2660公里。农田水利设施加快建设，实施高标准基本农田项目121.7万亩，新增耕地9.2万亩、建设用地2.79万亩，完成小型水库除险加固104座，新建和整治渠道2328公里。城乡环境明显改善。城乡环境综合治理成效明显，“五乱”“三线”有效治理，“大城管”“大环卫”工作格局全面构建，农村生活垃圾治理首批通过国家验收，东坡湖综合整治顺利完成，“龙鹄模式”眉山经验全国推广。“绿海明珠”“千湖之城”“百园之市”深入推进，森林覆盖率48.08 % ，城乡绿化覆盖率50.8 % ，新增水域面积80平方公里，新建和建成各类公园92个。东坡城市湿地公园、“四苏公园”赢得群众热烈点赞，眉山城市形象得到极大提升，眉山城市内涵得到极大丰富。洪雅县成功创建国家级生态县。国家园林城市、省级环境保护模范城市、省级文明城市成功创建。全市城市公共文明指数测评连续四年位居全省前3位。

坚持和谐发展，从基本民生走向幸福民生，社会环境更加良好。五年来，我们始终把人民对美好生活的向往作为奋斗目标，不断增进民生福祉，让群众有更多获得感。民生实事全面完成。解决121.6万农村人口饮水安全。完成棚户区改造31834户，解决住房困难群众9万人。实施农村危旧房改造39937户，完成投入27.2亿元。百万安居工程建设开工率、农村危房改造竣工率连续4年全省第一，地质灾害防治连续7年实现零伤亡。住房公积金管理率先在全国实现“贯标”。社会保障全面加强，城乡居民基本医疗保险制度有机整合，城乡低保标准进一步提高，社会养老服务体系加快建设，城镇居民“零就业”家庭保持动态为零，农村五保集中供养率连续五年全省第一。各项事业全面提升。教育事业加快发展，五年完成教育投入156亿元，新建和改扩建校舍130万平方米，义务教育招生改革经验全省推广，高校总数居全省第3位。卫生计生事业不断加强，医药卫生体制改革深入实施，基本药物制度实现全面覆盖，血吸虫病传播阻断全部达标，县（区）人民医院全部创建为二甲以上综合医院。公共文化服务体系日臻完善，文化立市“123”工程深入推进，“两宋荣光”眉州大道、东坡宋城I期顺利竣工，三苏祠灾后重建全面完成。成功举办中国泡菜展销会，成功申办第九届中国竹文化节，青神竹编产业园创建为国家级版权示范园。第九届残运会飞镖和盲人门球项目圆满承办。社会治理全面加强。依法治市“八大体系”深入实施，网格化服务管理全面推进，“平安眉山”建设成效明显，“大调解”体制机制和社会稳定风险评估机制深化完善，“六五”普法顺利通过省级验收，“诉非衔接”改革试点被中央改革办列为全国首批30个改革成功案例，眉山经验上升为国家经验。“维稳跟着项目走”工作模式全国推广，信访“三无”创建、网上信访和群众来访“四个当天”办理制度全面推行，进京到省上访总量连续八年全省末位。国防动员和后备力量建设全面加强，省级双拥模范城市成功创建。安全生产形势持续好转，无重大及以上安全事故发生。

坚持从严从实，从转变职能走向法治政府，自身建设更加有为。五年来，我们紧紧围绕“五型”政府建设这个核心抓手，不断革故鼎新、与时俱进，自身建设实现新突破。治理能力进一步增强。应急处突反应迅速，成功应对“4·20”芦山强烈地震、“7·9”特大暴雨洪灾、“6·13”映天学校公共卫生突发事件。27个纳入全省“4·20”芦山强烈地震灾后恢复重建总体规划的项目顺利竣工，65698户城乡居民住房重建、维修加固全面完成。依法行政深入实施，行政权力公开透明运行平台建成投运，制定出台各类规范性文件415份。行政效能进一步提高。深入推进行政审批制度改革，大力简政放权，下放和取消行政审批51项，转变管理方式81项。大力加强政务服务体系建设，率先在全国建立“三大服务中心”，率先在全省推进行政权力集中进中心，全市131个乡镇（街道）、1103个村（社区）、8487个村民小组全部建立便民服务中心和站点，五级便民服务体系不断完善。政务服务五星级管理做法被编入全国政务服务工作创新案例。认真办理人大代表建议、政协提案，满意和基本满意率达100 % 。作风建设进一步加强。扎实开展群众路线教育实践活动，深入推进“三严三实”专题教育，持续用力正风肃纪，大力整治“庸懒散浮拖”，“四风”问题得到有效遏制，干部作风持续好转。认真贯彻落实中央“八项规定”、省委“十项规定”、市委“六个坚持”，全面加强政府廉政建设，市本级“三公”经费下降34.86 % ，政府系统公文减少19.5 % 。

各位代表！

2015年，是实施“十二五”规划的最后一年，也是本届政府任期目标攻坚之年。面对严峻复杂的国内外形势和持续加大的经济下行压力，我们始终坚持在综合施策中稳增长，始终坚持在动能转换中调结构，始终坚持在包容共享中惠民生，经济运行“稳”的基础更加牢固，“转”的进程不断加快，“进”的态势积极向好。回顾一年的奋斗历程，虽然困难比预料的多，但结果比预想的好，我们顺利完成了市三届人大六次会议确定的各项目标任务。预计，全市GDP增长10 % ；全社会固定资产投资1020亿元，增长10.5 % ；地方一般公共预算收入83.1亿元，增长10.5 % ；规上工业增加值增长12 % ；社会消费品零售总额360亿元，增长12.5 % ；三产增加值增长10 % ；城乡居民人均可支配收入分别增长9 % 、10 % 。

五年拼搏不平凡，五年成就不寻常。“十二五”时期的接续奋斗和艰辛实践，不仅创造了巨大的物质财富，也创造了巨大的精神财富。成绩来之不易，这是省委、省政府和市委坚强领导的结果，是市人大和市政协监督支持的结果，是全市人民齐心协力、顽强拼搏的结果。在此，我谨代表市人民政府，向历任老领导、老同志，向全市人民、解放军驻眉部队和武警、消防官兵、政法干警，向关心、支持和参与眉山建设发展的社会各界朋友，表示衷心的感谢并致以崇高的敬意！

在肯定成绩的同时，我们也要清醒地看到发展中面临的困难和问题：经济总量不大、运行质量不高、产业结构不优，仍是眉山加快发展的最大障碍；重大产业项目缺乏、自主创新能力不强、资源环境约束加大，仍是眉山永续发展的最大瓶颈；城乡发展不平衡、区域发展不协调、公共服务不配套，仍是眉山均衡发展的最大短板；社会矛盾增多、社会治理难度加大、系统风险加剧，仍是眉山和谐发展的最大挑战；转变政府职能、规范行政行为、提高行政效率，仍是政府自身建设的最大主题。对上述问题，我们将高度重视，认真加以解决。
